# JNK activation dynamics drive distinct gene expression patterns over time mediated by mRNA stability

**DOI:** 10.1038/s41540-025-00590-2

**Published:** 2025-10-21

**Authors:** Abbas Jedariforoughi, Rachel Burke, Andrew Chesak, Jose L. Gonzalez Hernandez, Ryan L. Hanson

**Affiliations:** 1 Department of Biology and Microbiology, South Dakota State University, Brookings, SD USA; 2 Department of Agronomy, Horticulture, and Plant Sciences, South Dakota State University, Brookings, SD USA; 3 Division for Research and Economic Development, South Dakota State University, Brookings, SD USA

**Keywords:** Cell biology, Computational biology and bioinformatics, Systems biology

## Abstract

c-Jun N-terminal kinase (JNK) plays a major role in the regulation of cell death. Numerous studies have highlighted how the dynamics of this kinase dictate whether cells survive in response to cellular stress or induce cell death mechanisms. However, it remains less clear how these dynamics potentially contribute to downstream gene expression patterns through regulated transcription factors like c-Jun. To investigate this question, we used a treatment strategy with the JNK agonist anisomycin to drive specific temporal dynamics of JNK activation: sustained, transient, or pulsed activation, and assessed the impact on downstream gene expression patterns. We observed that multiple gene expression patterns emerged depending on the temporal dynamics of JNK activation. Ordinary differential equation (ODE) models suggest that a subset of these clusters is mediated by mRNA stability, a finding supported by experimental datasets of mRNA decay rates. Specific gene clusters also show enrichment in specific cellular pathways, including cell death and inflammatory signaling, suggesting that JNK dynamics contribute to differential regulation of these pathways. These findings highlight another contribution of JNK dynamics to the regulation of cellular responses to stress stimuli.

## Introduction

Cells maintain homeostasis by rapidly sensing and responding to internal and external changes in cell state. Cells employ multiple mechanisms to discriminate between different stimuli, for example, diverse receptors that distinguish between ligands^1–3^, selective activation of signaling molecules^4^, as well as variation in the temporal dynamics of the specific downstream pathways^5,6^. This process of “dynamic encoding” enables cells to differentiate between stimuli that converge upon the same signaling networks. For example, temporal dynamics of the stress-responsive transcription factor p53 allow cells to distinguish between ultraviolet (UV) light-induced damage, DNA double-strand breaks, as well as specific chemotherapies^7–11^. These dynamics give rise to specific gene and protein expression patterns^7,12–14^, as well as elicit specific cell fate outcomes^8,9^. Similarly, nuclear factor (NF)-κB exhibits stimulus-specific dynamics following exposures to pathogen-associated molecular patterns (PAMPs), damage-associated molecular patterns, and cytokines to drive unique gene expression patterns^3,15^. The precise gene expression dynamics elicited in the case of p53 and NF-κB rely on both the dynamics of the transcription factor itself and the mRNA stability of the specific target gene^6,7,12^.

Dynamic encoding is not strictly limited to transcription factors, as the mitogen-activated protein kinase (MAPK) c-Jun N-terminal kinase (JNK) has also been proposed to exhibit dynamic encoding as a means of regulating cell fate outcomes in response to cellular stress. Specifically, JNK mediates the distinction between pro-survival and pro-death signaling. Initial characterization of JNK’s function in the mid-1990s highlighted the potential role for the duration of JNK activation in mediating cell survival or apoptosis in response to UV light damage^16^. Similar approaches demonstrated a biphasic JNK response in mediating pro-survival or pro-death responses following induction of endoplasmic reticulum stress induced by tunicamycin or thapsigargin^17^. More recently, the use of fluorescent biosensors of JNK activation^18^ has facilitated the tracking of single-cell kinase activity and cell fates. These studies have highlighted roles for JNK dynamics in the regulation of cell death in response to UV light^19^, oxidative stress^20^, and activation of the inflammasome in pyroptosis^21^.

While these studies have emphasized clear roles of JNK dynamics in mediating cellular responses, the downstream regulatory impacts of these dynamics remain less clear. Unlike the transcription factors p53 and NF-κB, JNK exerts its effects by phosphorylation of serine and threonine residues on its nearly 100 substrates^22,23^. These include non-nuclear substrates Bcl2, Bax, Bim, and Bad, which may regulate cell fate independently of new gene expression. However, in addition to these non-nuclear substrates, JNK is also known to phosphorylate several transcription factors to regulate downstream gene expression patterns. The most well-characterized of these substrates is c-Jun, which is phosphorylated on the N-terminal residues serine 63 and serine 73 to potentiate its transcriptional activity as part of the dimeric transcription factor AP-1^22,23^. Currently, it is unclear how these dynamic patterns of JNK activation potentially contribute to downstream gene expression patterns.

In order to examine this question, we leveraged live-cell imaging approaches of fluorescent biosensors to track JNK activation within single cells. As JNK dynamics can be significantly heterogeneous between single cells and across treatments, we established a dosing regimen of anisomycin to enrich in specific dynamics, notably the duration and number of pulses, and assess gene expression impacts by RNA-seq. We found that cells exhibited an array of gene expression patterns in response to dynamic JNK activation. These dynamics can be partially explained by mRNA stability, similar to p53 and NF-κB, but other complex mechanisms are likely present, such as transcriptional cross-talk or variations in promoter affinity. Interestingly, certain gene clusters were enriched in specific pathways, suggesting that these dynamics may allow cells to diversify function over time in response to JNK activation. These results provide us with new insight into how JNK-mediated signal transduction can contribute to diverse cellular outcomes.

## Results

### JNK dynamics are diverse and heterogeneous across treatments

**Fig. 1:**
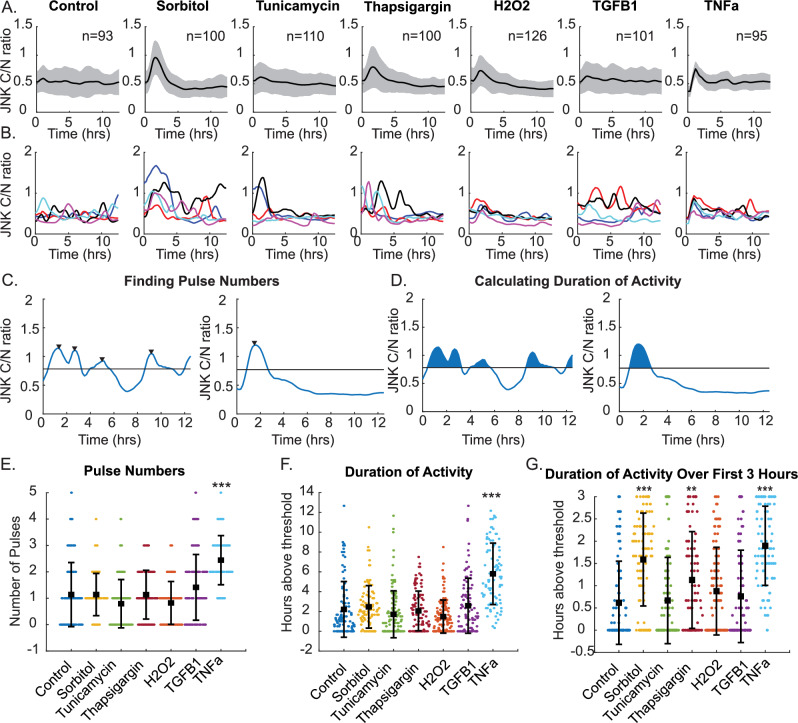
JNK dynamics vary in duration and pulse number across stimuli. **A** Average cytoplasmic to nuclear ratio of JNKKTR traces of RPE cells treated with DMSO control media, sorbitol (100 mM), tunicamycin (1 µM), thapsigargin (1 µM), H_2_O_2_ (50 µM), TGF-β1 (10 ng/ml), or TNFα (20 ng/ml) for 12.5 h. The thick line represents the average response, with the shaded area equal to the standard deviation. *n* represents the total number of analyzed cells. **B** Representative cell traces from five individual cells from the same treatment conditions, with each line representing the JNK C/N ratio. **C** Example traces identifying pulse numbers or **D** total duration of JNK activity within individual cells. **E** Plot of calculated pulse numbers for each condition. Average and standard deviation for each condition are shown. ***p < 0.001 as compared to control by ANOVA. **F** Total duration of activity for each individual cell. Bars show the mean ± SD. ***p < 0.001 as compared to control by ANOVA. **G** Duration of JNK activity over the first 3 h of treatment. Bars represent mean ± SD. **p < 0.01, ***p < 0.001 as compared to control by ANOVA.

Previous studies using live-cell imaging and western blot approaches have proposed distinct dynamics of JNK activation in response to diverse stimuli. To examine the emergence and heterogeneity of JNK dynamics, we performed live-cell imaging of RPE1-hTERT cells expressing the JNKKTR fluorescent biosensor to track single-cell JNK dynamics. We examined JNK activity in response to sorbitol (200 mM), tunicamycin (1 µM), thapsigargin (1 µM), H_2_O_2_ (50 µM), transforming growth factor-β1 (TGF-β1) (10 ng/ml), and tumor necrosis factor-α (TNFα) (20 ng/ml) treatment and analyzed 90+ individual cells per condition over 12.5 h. Sorbitol, thapsigargin, H_2_O_2_, and TNFα treatment all exhibited a visible response within the population with a rapid pulse of JNK activation (Fig. 1A). At the single-cell level, individual cells showed marked heterogeneity, with some cells responding and others not responding at all (Fig. 1B). To identify the dynamics that emerged in response to treatments, we identified the number of pulses of JNK activation by identifying local maxima (Fig. 1C) and the total duration of activation by quantifying the area under the curve above an assigned threshold of 50% above baseline (Fig. 1D). This baseline was established for each condition based on the average of four frames before addition of treatments. Overall, only TNFα treatment resulted in significant increases in the number of JNK pulses and activity over the 12.5-h imaging experiment. However, this is likely due to the fact that many treatments induced a singular pulse of activation followed by inactivation. Consistent with this, we observed that sorbitol, thapsigargin, and TNFα all showed significantly longer durations of JNK activation within the first three hours (Fig. 1G). Overall, within these experiments, we identify a number of dynamics among single cells in a population, including singular pulses, multiple pulses, as well as prolonged or sustained activation (Supplementary Fig. 1A–D) across all conditions. Consequently, we wanted to interrogate how these particular dynamics may contribute to downstream gene expression patterns.

### Anisomycin dosing can be used to induce specific dynamic patterns

**Fig. 2:**
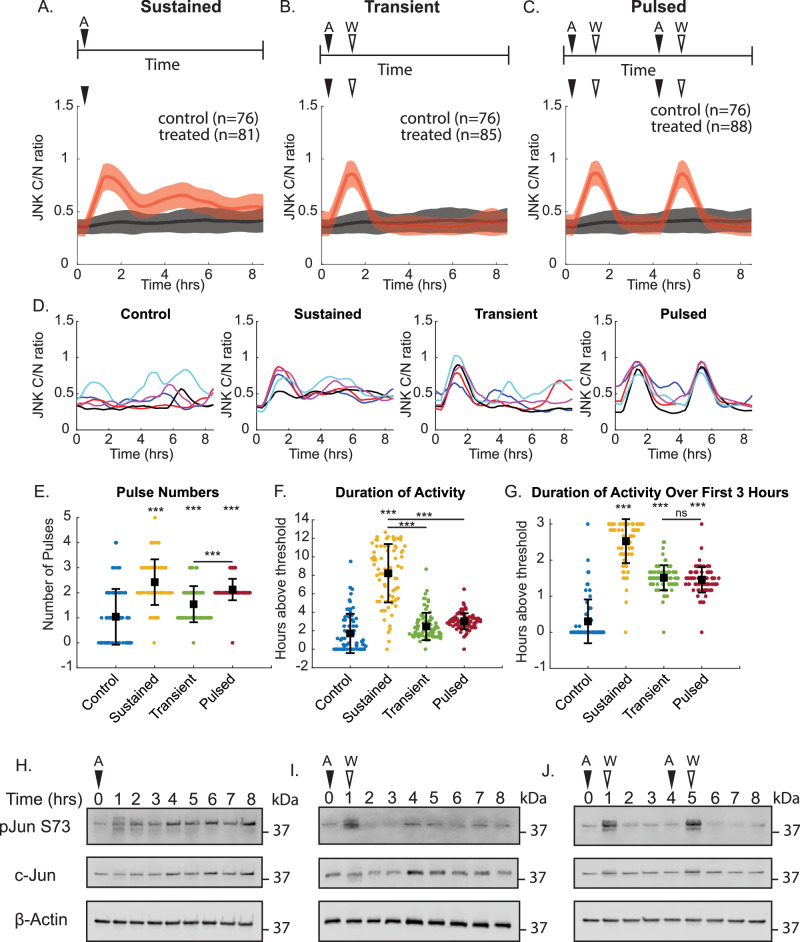
Anisomycin dosing scheme can enrich in specific dynamic patterns. **A**–**C** Average JNK C/N ratios of cells treated with sustained (**A**), transient (**B**), or pulsed (**C**) anisomycin. Treated cells are shown in red versus the DMSO vehicle control in black. Thick lines represent the mean response, with shaded areas equal to the standard deviation. *n* is the number of cells analyzed. **D** Representative traces of five individual cells from each treatment and control condition. **E** Pulse numbers for each individual cell over the duration of the imaging. Bars shown mean ± SD. ***p < 0.001 as assessed by ANOVA. Comparisons between treated and control groups unless otherwise indicated. **F** Total duration of JNK activity over the 8.5-h experiment. Bars show mean ± SD. ***p < 0.001 as assessed by ANOVA. Comparisons between treated and control groups unless otherwise indicated. **G** Duration of JNK activity over the first 3 h. Bars show the mean ± SD. ***p < 0.001 as assessed by ANOVA. *N*s = not significant. Comparisons between treated and control groups unless otherwise indicated. **H**–**J** Western blot images of phosphorylated c-Jun (pJun S73), total c-Jun, and β-actin in response to sustained (**H**), transient (**I**), or pulsed (**J**) anisomycin treatment confirming phosphorylation patterns of c-Jun mirror KTR traces.

A significant challenge in translating pathway activation dynamics to gene expression is desynchronization of the population over time, which masks overall gene expression patterns^12^. To overcome this limitation, we chose to utilize an approach with repeated dosing to enrich in specific dynamics (transient/singular pulse, multiple pulses, and sustained). However, stimuli like H_2_O_2_ and TNFα are known to induce cell death at high doses^20,24^. Likewise, tunicamycin and thapsigargin are both associated with biphasic JNK activation, making it difficult to induce sustained or pulsed dynamics^17^. Instead, we chose to dose cells with the JNK agonist anisomycin. As anisomycin can inhibit protein translation, we used a subinhibitory concentration of 50 ng/ml to minimize disruptions to protein synthesis while still inducing strong JNK activation^25^. By varying the timing of anisomycin application and removal, we were able to generate three distinct activation profiles: sustained activation, transient activation, and repeated pulses of activation as assessed by live-cell imaging and quantification of JNKKTR cytoplasmic-to-nuclear ratio compared to an untreated control group. Cells were imaged 30 min prior to the addition of anisomycin to establish a baseline of activity. In the sustained activation model, continuous exposure to anisomycin led to prolonged JNK activation (Fig. 2A). Conversely, transient activation was achieved by adding anisomycin followed by its rapid washout, resulting in a single, brief pulse of JNK activation (Fig. 2B). Lastly, repeated pulses of JNK activation were generated by alternating the addition and washout of anisomycin (Fig. 2C). At the single-cell level, this mechanism of repeated dosing resulted in strong synchronization of the two pulses (Fig. 2D). To confirm the anisomycin dosing was driving different dynamics as expected, we calculated overall pulse numbers and total duration of JNK activation over the full 8.5-h imaging experiment using the same approach as in Fig. 1. As expected, we observed that the pulsed cells primarily exhibited two clear pulses and, on average, more than the single pulse condition (Fig. 2E). Furthermore, the sustained condition, as anticipated, showed longer durations of JNK activation (Fig. 2F). Over the first three hours the transient and pulsed conditions were largely identical and showed no significant difference, as expected (Fig. 2G).

To confirm that JNKKTR dynamics were reflective of endogenous transcription factor activation, we performed western blot analysis for phosphorylation of c-Jun at serine 73 (Fig. 2H–J). Consistent with the KTR measurements, sustained activation of JNK was characterized by prolonged phosphorylation of c-Jun over the observed time points, beginning as early as 1-h post-stimulation and maintaining high levels up to eight hours (Fig. 2H). In the case of transient JNK activation, the western blot analysis revealed a rapid increase in c-Jun phosphorylation at serine 73, peaking at approximately 1-h post-stimulation, followed by a sharp decline as the pathway returned to baseline activity (Fig. 2I). Pulsed JNK activation exhibited periodic phosphorylation of c-Jun at serine 73 (Fig. 2J). Studies of the tumor suppressor p53 have shown that mRNA stability mediates gene expression patterns in response to pulsatile p53, and these predicted dynamics can be modeled using ordinary differential equations (ODEs). We therefore wanted to determine whether we could similarly predict JNK-dependent gene expression patterns using these modeling approaches.

### ODE model of JNK signaling predicts dynamic gene expression patterns

**Fig. 3:**
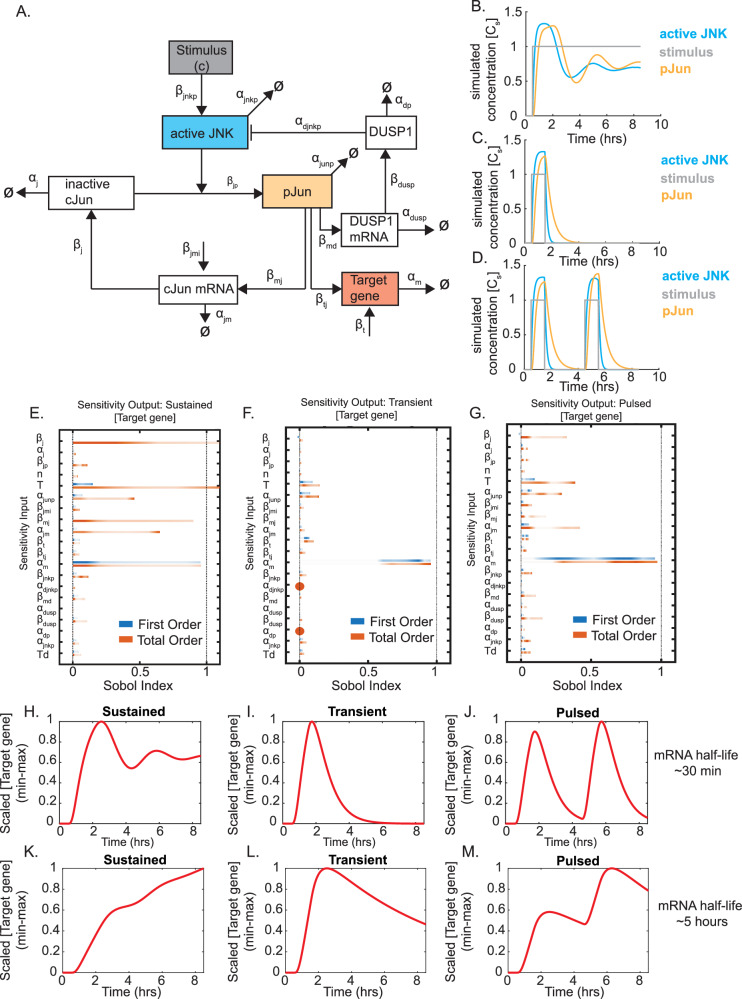
ODE model of JNK signaling predicts dynamic gene expression patterns. **A** Schematic representation of ODE model of JNK signaling with arrows representing production terms and Ø representing degradation. **B**–**D** Simulations of active JNK (blue), phosphorylated c-Jun (orange), and stimulus (gray) for sustained (**B**), transient (**C**), or pulsed (**D**) stimuli. Data shows plots of expression in simulated concentration units (*C*_s_) for each component over time (hours). **E**–**G** Global sensitivity analysis of target gene output relative to all model parameters across sustained (**E**), transient (**F**), and pulsed (**G**) model conditions. First-order and total order Sobol indexes are plotted for each parameter, with the darker color indicating more recurrent values. mRNA degradation rates (*α*_m_) exhibited the highest first-order index and thus were the primary driver of target gene variance. **H**–**M** Simulated gene expression patterns of target genes for sustained, transient, or pulsed JNK activation with either a short or long-lived mRNA over time (hours). Data was scaled by min–max normalization between 0 and 1.

To predict how JNK dynamics may influence gene expression patterns, we employed a simple ODE model (“Methods”—Tables 1 and 2) based on known regulatory factors within the JNK signaling cascade (Fig. 3A). This model was defined by seven equations.

1 $$\begin{array}{lll}\dfrac{d[inactive\,cJun]}{dt}&=&{\beta }_{j}\left[cJun\,mRNA\right]-{\alpha }_{{j}}[inactive\,cJun]\\&&-\,{\beta }_{{j}{p}}\left[inactive\,cJun\right]\dfrac{{\left[active\,JNK\right]}^{{n}}}{{\left[active\,JNK\right]}^{{n}}+{T}^{{n}}}\end{array}$$

The simulated concentration (*C*_s_) of inactive c-Jun (i.e., non-phosphorylated) depends linearly on the translation of c-Jun mRNA (*β*_j_), degradation of c-Jun protein (*α*_j_), and phosphorylation via active JNK (*β*_jp_). The half-life of c-Jun has been estimated between 70 and 90 min^26^, and *α*_j_ was set to 0.5 h^−1^. Phosphorylation rate was modeled as a Hill equation based upon previous models of p53 dynamics via ATM phosphorylation^10,11^. The Hill coefficient (*n* = 4) is consistent with estimates from previous studies of JNK activity^27^, and the half-maximal concentration (*T* = 1 *C*_s_) was based on prior models of p53 dynamics^10,11^. *β*_j_ and *β*_jp_ were arbitrarily assigned equal values of 3 h^-1^. Equal values were used to ensure total c-Jun did not vary dramatically over time, as we observed minimal effects on overall c-Jun levels in Fig. 2J. Varying this arbitrary rate had minimal impacts on the qualitative dynamics of target gene output as long as *β*_j_ and *β*_jp_ were kept equal but did impact the absolute quantification of target mRNA (Supplementary Fig. 2A).

2 $$\frac{{\rm{d}}\left[\mathrm{pJun}\right]}{{\rm{d}}t}={\beta }_{\mathrm{jp}}\left[\mathrm{inactive}\,\mathrm{cJun}\right]\frac{{\left[\mathrm{active}\,\mathrm{JNK}\right]}^{n}}{{\left[\mathrm{active}\,\mathrm{JNK}\right]}^{n}+{T}^{n}}-{a}_{\mathrm{junp}}\left[\mathrm{pJun}\right]$$

Phosphorylation of c-Jun (pJun) was computed using a Hill function to approximate phosphorylation of inactive c-Jun with parameters as defined in Eq. (1). The degradation rate of pJun (*α*_junp_) was estimated as 2.1 h^−1^, corresponding to a half-life of approximately 20 min based on rapid loss of phosphorylation within 1 h in transient and pulsed conditions (Fig. 2J).

3 $$\frac{{\rm{d}}\left[\mathrm{cJun}\,\mathrm{mRNA}\right]}{{\rm{d}}t}={\beta }_{\mathrm{jmi}}+{\beta }_{\mathrm{mj}}\left[\mathrm{pJun}\right]-{a}_{\mathrm{jm}}\left[\mathrm{cJun}\,\mathrm{mRNA}\right]$$

Changes in c-Jun mRNA over time were defined by basal transcription rate (*β*_jmi_) and autoregulation via pJun (*β*_mj_)^28^. These were arbitrarily assigned values of 1 *C*_s_ h^−1^ and 1.5 h^−1^, respectively. Varying the strength of autoregulation had relatively minor impacts on the dynamics of target gene expression (Supplementary Fig. 2B); however, increasing the strength of autoregulation dramatically increased overall levels of c-Jun (Supplementary Fig. 2C), which did not align with the results from Fig. 2J, thus we assumed a modest autoregulation value. Degradation of c-Jun transcripts has been estimated at 20 min^29^, and therefore we assigned *α*_jm_ a value of 2.1 h^−1^.

4 $$\frac{{\rm{d}}\left[\mathrm{Target}\,\mathrm{Gene}\right]}{{\rm{d}}t}={\beta }_{{\rm{t}}}+{\beta }_{\mathrm{tj}}\left[\mathrm{pJun}\right]-{a}_{{\rm{m}}}\left[\mathrm{Target}\,\mathrm{Gene}\right]$$

Changes in target gene expression over time were similarly defined by a basal transcription rate (*β*_t_) and pJun-dependent transcription rate (*β*_tj_) along with a turnover rate (*α*_m_). To specifically simulate c-Jun-dependent genes, we assigned *β*_tj_ > *β*_t_. For our included model, these values were set at 3 h^−1^ and 1 *C*_s_ h^−1^, respectively; however, while varying the strength of pJun-dependent transcription (*β*_tj_) impacts the magnitude of simulated expression (*C*_s_), it has no impact on the qualitative shape of dynamics (Supplementary Fig. 2D).

5 $$\frac{{\rm{d}}\left[\mathrm{active}\,\mathrm{JNK}\right]}{{\rm{d}}t}={\beta }_{\mathrm{jnkp}}\left[c\right]-{a}_{\mathrm{jnkp}}\left[\mathrm{active}\,\mathrm{JNK}\right]-{a}_{\mathrm{djnkp}}\left[\mathrm{active}\,\mathrm{JNK}\right]\frac{{\left[\mathrm{DUSP}1\right]}^{n}}{{\left[\mathrm{DUSP}1\right]}^{n}+{\mathrm{Td}}^{n}}$$

Activation of JNK was modeled using a production rate (*β*_jnkp_) linearly related to a stimulus (*c*), which was modeled as a step function corresponding to either a zero for inactive or one for active stimulation. Inactivation of JNK was dependent on turnover (*α*_jnkp_) and dephosphorylation (α_djnkp_) through the phosphatase DUSP1^22^. Parameter values for *β*_jnkp_, *α*_jnkp_, *α*_djnkp_, *n*, and Td were based upon previous models of ATM activation and regulation in p53 dynamic models^10,11^, which exhibit similarly rapid inactivation between pulses.

6 $$\frac{{\rm{d}}\left[\mathrm{DUSP}1\,\mathrm{mRNA}\right]}{{\rm{d}}t}={\beta }_{\mathrm{md}}\left[\mathrm{pJun}\right]-{\alpha }_{\mathrm{dusp}}\left[\mathrm{DUSP}1\,\mathrm{mRNA}\right]$$

Changes in the mRNA of DUSP1 were modeled using a pJun-dependent production rate (*β*_md_) and mass-action turnover rate (*α*_dusp_). *β*_md_ production was set at 0.25 h^-1^ based on similar model dynamics of negative feedback in p53 models^10,11^. DUSP1 mRNA turnover has been estimated broadly from 15 min to upwards of 1 h^30,31^ and *α*_dusp_ was set at 2.1 h^−1^, approximating a half-life of 20 min. Varying production rates or increasing stability of DUSP1 mRNA results in the formation of dampened pulses of pJun in both pulsed and sustained conditions that do not align with either KTR results or western blot analysis in Fig. 2 (Supplementary Fig. 2E).

7 $$\frac{{\rm{d}}\left[\mathrm{DUSP}1\right]}{{\rm{d}}t}={\beta }_{\mathrm{dusp}}\left[\mathrm{DUSP}1\,\mathrm{mRNA}\right]-{\alpha }_{\mathrm{dp}}\left[\mathrm{DUSP}1\right]$$

Changes in protein levels of DUSP1 were modeled using mass-action equations driven by translation rate of DUSP1 mRNA (*β*_dusp_) and degradation of DUSP1 (*α*_dp_). Half-lives of DUSP1 have been estimated between 40 min and 2 h^32,33^. For this model we assigned a degradation rate of 0.693 h^−1^, approximating a half-life of 1 h. Production rates were arbitrarily set at 1 h^−1^. As with DUSP1 mRNA, increasing production rates or stability resulted in loss or dampening of secondary pulses that did not fit with either KTR or western blot results in Fig. 2 (Supplementary Fig. 2F). Decreasing production rates or stability resulted in a second pulse of phosphorylation greater than the first, which also did not align with the KTR results, therefore, *α*_dp_ and *β*_dusp_ were kept at 0.693 h^−1^ and 1 h^−1^, respectively.

**Table 1:** ODE equations used in the model

Number	Equation
**(1)**	$$\begin{array}{l}\frac{{\rm{d}}\left[\mathrm{inactive}\,\mathrm{cJun}\right]}{{\rm{d}}t}={\beta }_{{\rm{j}}}\left[\mathrm{cJun}\,\mathrm{mRNA}\right]-{\alpha }_{{\rm{j}}}\left[\mathrm{inactive}\,\mathrm{cJun}\right]-{\beta }_{\mathrm{jp}}\left[\mathrm{inactive}\,\mathrm{cJun}\right]\frac{{\left[\mathrm{active}\,\mathrm{JNK}\right]}^{n}}{{\left[\mathrm{active}\,\mathrm{JNK}\right]}^{n}+{T}^{n}}\end{array}$$
**(2)**	$\frac{{\rm{d}}\left[\mathrm{pJun}\right]}{{\rm{d}}t}={\beta }_{\mathrm{jp}}\left[\mathrm{inactive}\,\mathrm{cJun}\right]\frac{{\left[\mathrm{active}\,\mathrm{JNK}\right]}^{n}}{{\left[\mathrm{active}\,\mathrm{JNK}\right]}^{n}+{T}^{n}}-{\alpha }_{\mathrm{junp}}\left[\mathrm{pJun}\right]$
**(3)**	$\frac{{\rm{d}}\left[\mathrm{cJun}\,\mathrm{mRNA}\right]}{{\rm{d}}t}={\beta }_{\mathrm{jmi}}+{\beta }_{\mathrm{mj}}\left[\mathrm{pJun}\right]-{\alpha }_{\mathrm{jm}}\left[\mathrm{cJun}\,\mathrm{mRNA}\right]$
**(4)**	$\frac{{\rm{d}}\left[\mathrm{Target}\,\mathrm{Gene}\right]}{{\rm{d}}t}={\beta }_{{\rm{t}}}+{\beta }_{\mathrm{tj}}\left[\mathrm{pJun}\right]-{\alpha }_{{\rm{m}}}\left[\mathrm{Target}\,\mathrm{Gene}\right]$
**(5)**	$\frac{{\rm{d}}\left[\mathrm{active}\,\mathrm{JNK}\right]}{{\rm{d}}t}={\beta }_{\mathrm{jnkp}}\left[c\right]-{\alpha }_{\mathrm{jnkp}}\left[\mathrm{active}\,\mathrm{JNK}\right]-{\alpha }_{\mathrm{djnkp}}\left[\mathrm{active}\,\mathrm{JNK}\right]\frac{{\left[\mathrm{DUSP}1\right]}^{n}}{{\left[\mathrm{DUSP}1\right]}^{n}+{\mathrm{Td}}^{n}}\,$
**(6)**	$\frac{{\rm{d}}\left[\mathrm{DUSP}1\,\mathrm{mRNA}\right]}{{\rm{d}}t}={\beta }_{\mathrm{md}}\left[\mathrm{pJun}\right]-{\alpha }_{\mathrm{dusp}}\left[\mathrm{DUSP}1\,\mathrm{mRNA}\right]$
**(7)**	$\frac{{\rm{d}}\left[\mathrm{DUSP}1\right]}{{\rm{d}}t}={\beta }_{\mathrm{dusp}}\left[\mathrm{DUSP}1\,\mathrm{mRNA}\right]-{\alpha }_{\mathrm{dp}}\left[\mathrm{DUSP}1\right]$

**Table 2:** Parameter definitions for the ODE model

Parameter	Name	Assumed model value	Sensitivity analysis range
*β*_j_	c-Jun protein production rate	3 h^−1^	2–4 h^−1^
*α*_j_	c-Jun protein degradation rate	0.5 h^−1^ (^26^)	0.3–1 h^−1^
*β*_jp_	Saturating production rate of pJun	3 h^−1^	2–4 h^−1^
*n*	Hill coefficient of c-Jun phosphorylation by active JNK	4 (^10,11,27^)	1–10
*T*	Active JNK concentration for half-maximal phosphorylation of c-Jun	1 *C*_s_ (^10,11^)	0.1–2 *C*_s_
*α*_junp_	pJun degradation rate	2.1 h^−1^	0.5–3 h^−1^
*β*_jmi_	Basal c-Jun mRNA production rate	1 *C*_s_ h^−1^	0.1–2 *C*_s_ h^−1^
*β*_mj_	pJun-dependent c-Jun mRNA production rate	1.5 h^−1^ (^28^)	1–3 h^−1^
*α*_jm_	c-Jun mRNA turnover rate	2.1 h^−1^ (^29^)	0.3–3 h^−1^
*β*_t_	Basal target gene mRNA production rate	1 *C*_s_ h^−1^	0.1–2 *C*_s_ h^−1^
*β*_tj_	pJun-dependent target gene mRNA production rate	3 h^−1^	2–4 h^−1^
*α*_m_	Target gene degradation rate	Variable dependent on figure (0.02 h^−1^–1.4 h^−1^)	0.02–1.4 h^−1^
*β*_jnkp_	Active JNK production rate	10 *C*_s_ h^−1^ (^10,11^)	5–15 *C*_s_ h^−1^
*α*_jnkp_	Active JNK degradation rate	7.5 h^−1^ (^10,11^)	3–10 h^−1^
*α*_djnkp_	Saturating dephosphorylation rate of Active JNK	50 h^−1^ (^10,11^)	30–70 h^−1^
Td	DUSP1 concentration for half-maximal JNK dephosphorylation	0.2 *C*_s_ (^10,11^)	0.01–1 h^–1^
*β*_md_	pJun-dependent DUSP1 mRNA production rate	0.25 h^−1^ (^10,11^)	0.1–2 h^−1^
*c*	Stimulus	0 or 1	NA
*α*_dusp_	DUSP1 mRNA degradation rate	2.1 h^−1^ (^30,31^)	0.3–3 h^−1^
*β*_dusp_	DUSP1 protein production rate	1 h^−1^	0.1–2 h^−1^
*α*_dp_	DUSP1 protein degradation rate	0.693 h^−1^ (^32,33^)	0.3–2.1 h^−1^

Using a simple step function for the stimulus, we could effectively simulate a sustained (Fig. 3B), transient (Fig. 3C), or pulsed stimulus (Fig. 3D) and predict active JNK and phosphorylated c-Jun levels, which qualitatively captured the experimentally observed dynamics of JNK activation from our single-cell imaging results (Supplementary Fig. 3A–C). As we wanted to predict how JNK dynamics impacted gene expression patterns, we performed a global sensitivity analysis of our model for target gene expression using the SimBio toolbox in MATLAB to compute first-order and total Sobol indexes for all parameters within the model across a range of parameter settings (“Methods”—Table 1), specifically focusing on the output of target gene expression to identify which parameters had the greatest impact on gene expression (Fig. 3E–G). These results indicated that across all three conditions (sustained, transient, or pulsed), the most critical parameter involved in target gene variance was the mRNA stability. This finding was consistent with established principles from other dynamic systems, such as p53 and NF-κB^6,12^, where mRNA stability plays a crucial role in determining the timing and magnitude of gene expression. By coupling our model with a downstream transcriptional output, we were able to predict gene expression profiles for short-lived (half-lives = 30 min) and long-lived (half-lives = 5 h) mRNAs under sustained, transient, and pulsed JNK activation (Fig. 3H–M). Specifically, we hypothesized that short-lived mRNAs would exhibit rapid changes in expression, closely mirroring the JNK activation pattern (Fig. 3H–J), while long-lived mRNAs would show more integrated responses, reflecting cumulative JNK activity over time (Fig. 3K–M). Based on these predicted gene expression patterns, we wanted to determine what biological patterns would emerge and whether the model could accurately predict these dynamic patterns.

### RNA sequencing reveals gene expression dynamics driven by JNK activation patterns

**Fig. 4:**
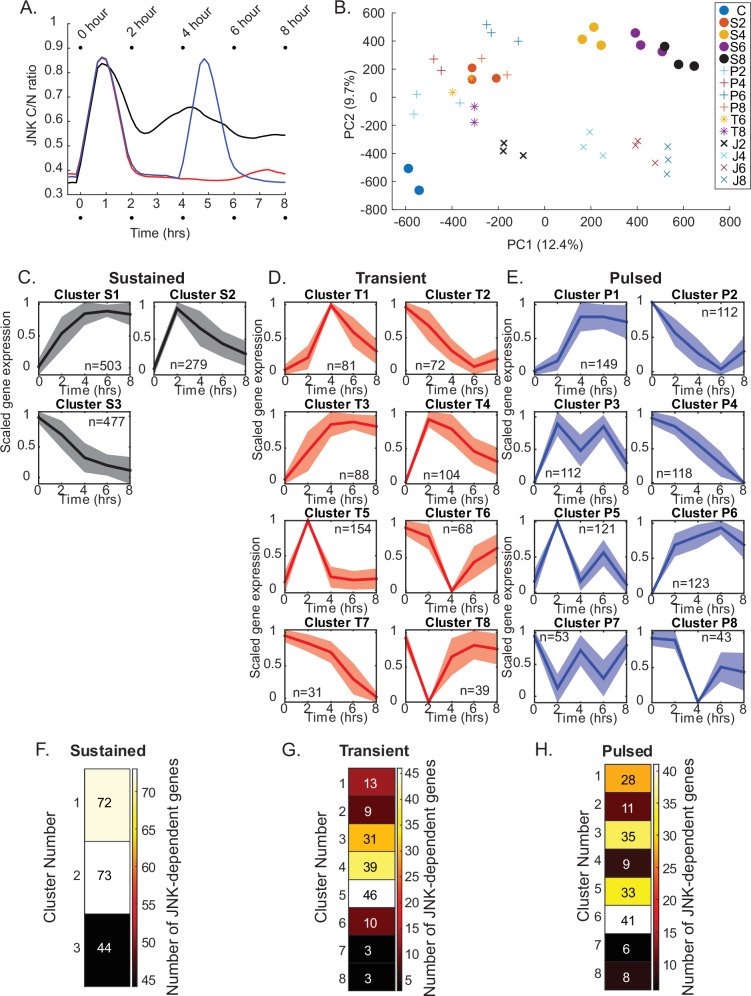
RNA-seq identifies gene expression clusters unique for specific patterns of JNK activation. **A** Schematic highlighting the time points where mRNA was collected. Data traces are mean traces from Fig. 2A–C used to highlight relationship of mRNA timepoints to JNK activity. **B** Principal Component Analysis (PCA) of RNA-seq timepoints and samples. C control, S sustained, T transient, P pulse. Numbers represent the specific time point. **C**–**E** K-means clusters of gene expression patterns for sustained (**C**), transient (**D**), or pulsed (**E**) JNK activity. Data shown as the mean ± SD of scaled expression data (0–1). *N* represents the number of genes within each cluster. **F**–**H** Number of JNK-dependent genes identified within sustained (**F**), transient (**G**), or pulsed (**H**) clusters.

In order to experimentally determine whether the predicted gene expression patterns emerged in cells exhibiting specific JNK dynamics, we treated cells with our dosing regimen as before and collected mRNA at 0, 2-, 4-, 6-, or 8-h post-treatment. These time points specifically lagged peak JNK activation from our KTR data (Fig. 4A) and c-Jun phosphorylation (Fig. 2H–J) by 1 h, as previous studies in p53 demonstrated peak gene expression lagging transcription factor activation by approximately 1 h^7,12^. As the first pulse of JNK is largely identical between pulsed and transient conditions (Fig. 2G), we used the same RNA for these conditions for the 2- and 4-h time points. In addition to the dynamic treatment groups, we also included an additional group which received sustained anisomycin treatment in the presence of the JNK inhibitor tanzisertib (10 µM). Preliminary analysis of the data identified five samples that exhibited poor read quality and were discarded from final analysis. Principal component analysis (PCA) was conducted to visualize the variance in gene expression profiles under sustained, transient, and pulsed JNK activation conditions (Fig. 4B). The PCA plot revealed distinct clustering of samples corresponding to each activation pattern, with PC1 and PC2 seeming to correlate with time and JNK activation, respectively.

To identify dynamic gene expression patterns across JNK dynamics, we first identified differentially expressed genes (DEGs) across multiple time points (2-, 4-, 6-, and 8-h post-treatment) for each activation pattern. If a gene was significantly up- or down-regulated at any of the time points, it was included for subsequent analysis. Unsurprisingly, the majority of DEGs were shared across conditions (Supplementary Fig. 4A). Fold change differences were scaled using min–max normalization to avoid clustering genes based on absolute differences in expression, followed by k-means clustering to group genes exhibiting similar expression dynamics over time (Supplementary Data 1). The clustering analysis revealed distinct gene expression profiles corresponding to each JNK activation pattern (Fig. 4C–E). Under sustained JNK activation, we observed predominantly sustained gene expression clusters, along with a transient cluster that peaked and then declined. Transient JNK activation induced clusters that showed rapid peaks in expression (Fig. 4D, clusters 4 and 5), followed by a swift return to baseline. However, other clusters exhibited sustained gene expression (Fig. 4D, cluster 3). Pulsed activation of JNK resulted in a few clusters that showed pulsatile gene expression to varying degrees (Fig. 4E, clusters 3 and 5). However, as before, other clusters showed more complex dynamics. Interestingly, many of the genes within a given cluster were highly conserved in other clusters when the dynamics of JNK changed. For example, transient cluster 5 and pulsed cluster 5 share about 90% of the identified genes (Supplementary Fig. 4B).

As anisomycin can activate additional pathways beyond JNK, we wanted to validate which clusters contained seemingly JNK-dependent genes. To identify these genes, we compared DEGs (log_2_ fold change greater than 1) between our sustained condition and our JNKi treatment group, which received both anisomycin and a JNK inhibitor. These JNK-dependent genes were then identified based on which cluster they occupied across conditions (Fig. 4F–H). As anticipated based on c-Jun’s role as a transcriptional activator, most JNK-dependent genes appeared within the upregulated gene clusters. In the case of transient JNK activation, these genes preferentially fell within clusters 3, 4, and 5 (Fig. 4G), whereas in response to pulsed activation JNK-dependent genes appeared in clusters 1, 3, 5, and 6 (Fig. 4H). Previous studies of p53 dynamics have proposed that diversification of gene expression patterns allows for potential differential regulation of specific pathways over time^12^. We hypothesized that perhaps JNK dynamics facilitated a similar process.

### Dynamic gene clusters are enriched in specific pathways

**Fig. 5:**
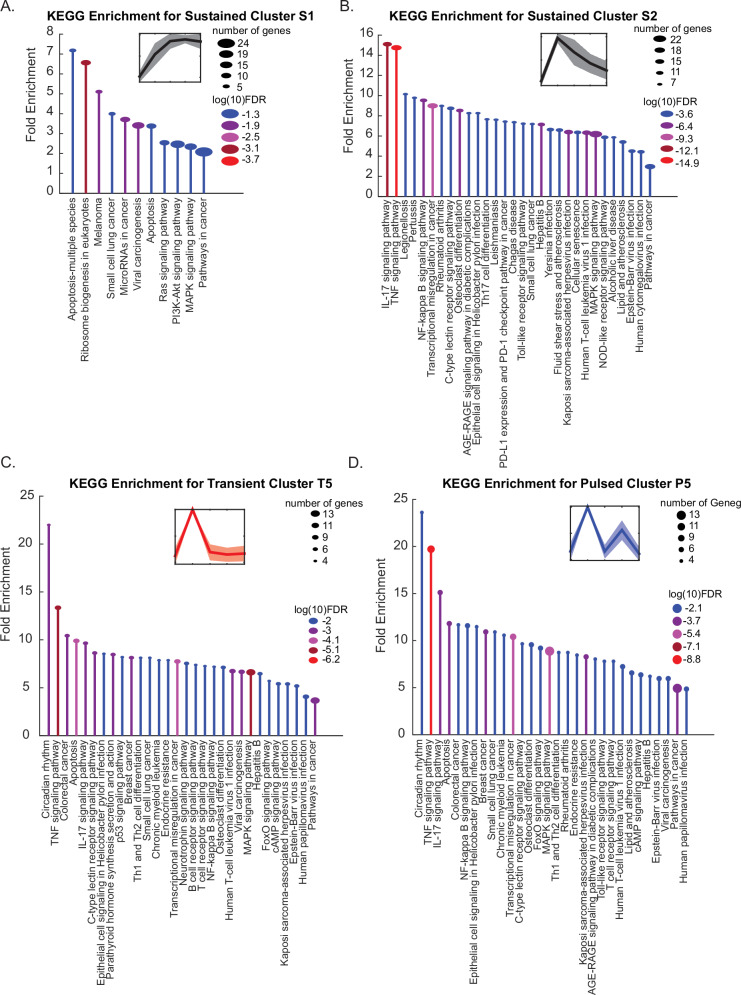
Gene expression clusters are associated with unique cellular signaling pathways. **A**–**D** Stem plots for enriched KEGG pathways for different dynamic clusters. **A** Sustained cluster 1. **B** Sustained cluster 2. **C** Transient cluster 5. **D** Pulsed cluster 5. The color of each stem correlates to the log10 FDR value, with the ball size correlating to the number of genes identified. KEGG pathway names are plotted below each stem. Data generated using ShinyGO 0.82 with plots prepared using MATLAB.

To explore the potential biological functions of these gene expression clusters, we performed functional enrichment analysis using the ShinyGO v0.82^34^. While not all gene clusters showed significant enrichment of KEGG pathways, we did observe several that were enriched in specific pathways. For example, sustained cluster 1 exhibited enrichment in a number of apoptosis- or cell death-associated proteins (Fig. 5A), including several pro-apoptotic factors such as *PMAIP1* (Noxa) and *BAK1* (Bak), suggesting prolonged activation of JNK may favor accumulation of these pro-apoptotic signals. In contrast, sustained cluster 2, which exhibits only a transient increase in gene expression, was enriched in several inflammatory signaling pathways, NF-κB response, and MAPK-associated responses (Fig. 5B). These results suggest that certain genes may be capable of persistence detection, for example remaining expressed in response to sustained stimulus, while other genes, like those in sustained cluster 2, may only be responsive to the initial stimulation or may require repeated pulses of stimulation. Interestingly, as many of the genes identified across dynamics are shared, similar enriched pathways were found across sustained, pulsed, or transient clusters. However, these pathways exhibited different expression dynamics depending on the pattern of JNK activation.

Sustained cluster 1 (Fig. 5A), transient cluster 5 (Fig. 5C), and pulsed cluster 5 (Fig. 5D) all showed enrichment of genes associated with apoptosis, but their expression patterns were distinct. Sustained cluster 1 showed sustained expression over time, while transient cluster 5 only showed a transient increase in expression. Pulsed cluster 5 exhibited two pulses of expression. These results suggest that perhaps the prolonged duration of JNK signaling may be necessary to maintain elevated expression of cell fate regulators involved in the induction of cell death. We hypothesized that, given the sustained upregulation or repeated pulses of apoptotic genes seen with sustained or pulsed JNK expression, we would observe increased cell death under these conditions. However, we observed no increase in sub-G1 cells as assessed by flow cytometry, though we did see a slight increase in G2 accumulation in response to sustained or pulsed JNK activation (Supplementary Fig. 5). This suggests additional mechanisms may be required for induction of cell death. Our ODE model predicts that these dynamics should be dictated by the mRNA stability of the specific target genes, and we wanted to examine whether this was indeed a regulatory mechanism involved.

### mRNA stability explains the dynamics of select clusters

**Fig. 6:**
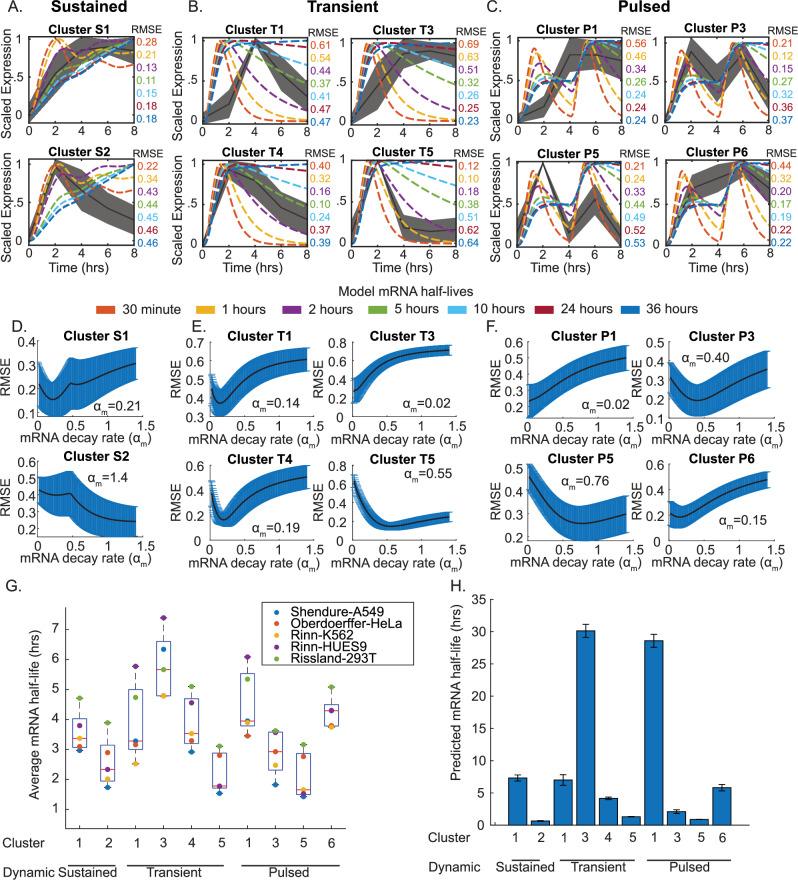
mRNA stability contributes to gene expression clusters driven by JNK activation. **A**–**C** Predicted scaled mRNA expression from our ODE model of JNK signaling was aligned to experimentally measured gene expression clusters for sustained (**A**), transient (**B**), or pulsed (**C**) JNK activity. Colored lines represent predicted mRNA expression for variable mRNA half-lives, with black lines showing experimentally measured gene expression dynamics. Root mean square error (RMSE) was computed for each prediction and shown to the right of each plot. **D**–**F** A parameter scan across 200 mRNA decay rates (*α*_m_) from 0.02 h^−1^ to 1.4 h^−1^ was conducted across all genes, and the average root mean square error was computed. Data plots show the average RMSE of all genes ±SD, with the minimal point being the predicted decay rate for each cluster. **G** Box plots showing average mRNA half-lives from multiple studies for genes within each identified cluster. Individual colors represent the average mRNA half-life from each study. **H** Bar graphs show model predictions for the average half-life of all genes within each cluster ±SEM.

To examine whether mRNA stability was predictive of our observed dynamic clusters, we compared the experimentally derived gene expression clusters with the predicted patterns from our ODE model with variable mRNA half-lives (Fig. 6A–C). Specifically, we focused strictly on the upregulated gene clusters based upon our model assumptions of c-Jun as a transcriptional activator and consistent with the majority of JNK-dependent genes residing within upregulated clusters (Fig. 4F–H). Calculation of deviation from the model by root mean square error (RMSE) suggested that several clusters fit better to specific mRNA stabilities. This included sustained cluster 1, transient clusters 4 and 5, as well as pulsed cluster 3. To estimate the predicted decay rates (α_m_), we expanded our parameter scan, testing 200 *α*_m_ values ranging from 0.02 to 1.4 h^−1^, corresponding to estimated half-lives of 36 h to 30 min, and identifying the decay rate with the minimal RMSE (Fig. 6D–F). Results suggest that clusters S1, T1, T3, T4, P1, and P6 correspond to long-lived mRNA, while S2, T5, P3, and P5 correspond to short-lived mRNA. However, certain clusters still showed relatively poor alignment with higher RMSE values, like cluster T1, suggesting potential additional regulatory mechanisms.

To determine whether our predictions aligned with measured mRNA half-lives, we examined multiple published datasets of global mRNA stability^35–38^. From these studies, we collected mRNA half-life measurements and cross-referenced these genes with the genes within each of our experimental clusters. For each study, we calculated the average mRNA half-life of genes within each cluster. One limitation of this approach is that not all genes appear in every dataset. However, based on this analysis, we found that many of the predictions aligned with experimental measurements (Fig. 6G). Genes within S2 do have shorter mRNA half-lives than genes from cluster S1 in these studies. Similarly, clusters T4 and T5 corresponded to longer-lived and short-lived mRNA, respectively. Likewise, clusters P3 and P5 also corresponded to short-lived mRNA, as predicted. While these clusters were supportive of mRNA stability as a driver, further investigation is needed to identify other major determinants of the observed dynamics for other clusters. As an additional measure of model agreement, we attempted to compute the predicted mRNA half-lives for all genes within each cluster (Fig. 6H). Overall, the trend remained similar to measured mRNA values in Fig. 6G; however, the model had a tendency to overestimate the stability of certain clusters, notably cluster T3 and P1.

### Hill function parameter analysis suggests promoter affinity contributes to gene clusters

While our initial model used a linear mass action term for target gene expression, many studies have used Hill functions to model transcriptional responses^39^. An advantage of this approach is the possibility of modeling relative affinities to promoters. To test whether our predictions could be improved, we replaced the linear mass-action expression of target genes with a Hill function, replacing Eq. 4 with the new equation.

8 $$\frac{{\rm{d}}\left[\mathrm{Target}\,\mathrm{Gene}\right]}{{\rm{d}}t}={\beta }_{{\rm{t}}}+{\beta }_{\mathrm{tj}}\frac{{\left[\mathrm{pJun}\right]}^{n}}{{K}^{n}+{\left[\mathrm{pJun}\right]}^{n}}-{\alpha }_{{\rm{m}}}\left[\mathrm{Target}\,\mathrm{Gene}\right]$$

**Fig. 7:**
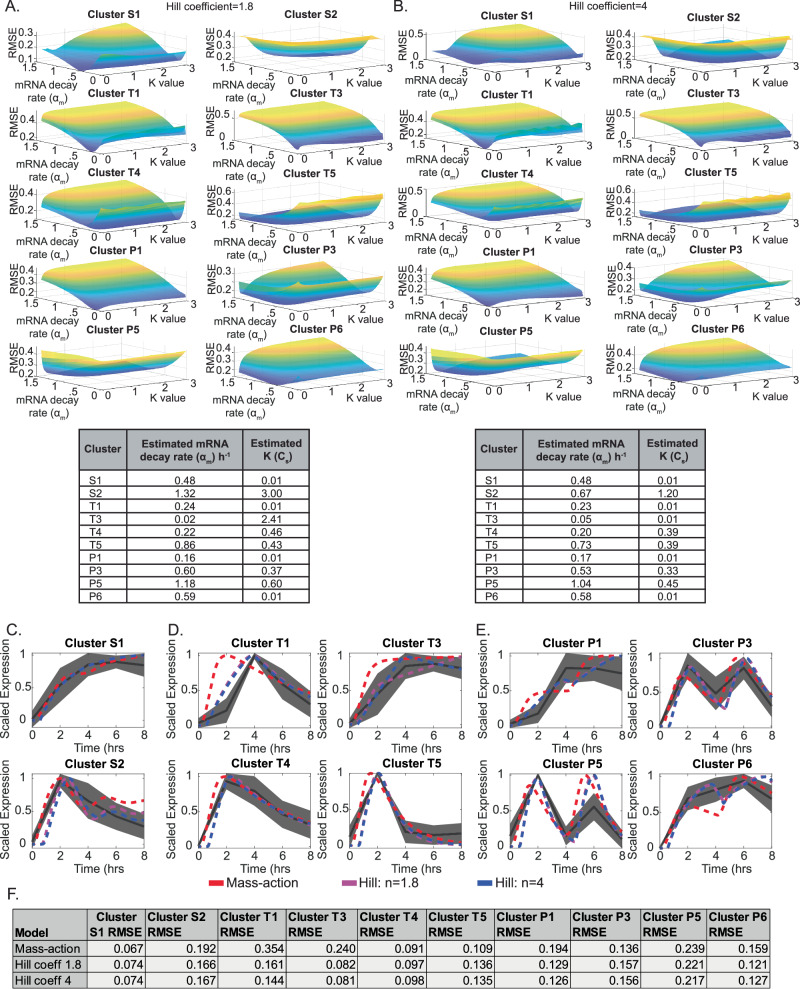
Hill function modeling of target gene expression suggests variation in promoter affinity. **A**, **B** Surface plots comparing the fit of the model to experimental gene expression by root mean square error (RMSE) across a 200 × 200 parameter scan of mRNA decay rates (*α*_m_) between 0.02 and 1.4 h^−1^, and *K* values from 0.01–3 *C*_s_. Minimum RMSE was used to calculate estimated decay rates and K values for each cluster, as shown in the table beneath each panel. **C**–**E** Comparison of the mass-action (red), Hill coefficient of 1.8 (magenta), and Hill coefficient of 4 (blue) models to experimental gene expression patterns (black) over time. **F** Computed root mean square error (RMSE) for each model as compared to each gene cluster.

*K* represents the concentration of pJun required for half-maximal gene expression. By modeling a low *K*, we can assume high affinity (i.e., low levels of pJun can strongly drive transcription), whereas high *K* values will require higher levels of phosphorylated c-Jun to drive gene expression. To our knowledge, the Hill coefficient of AP-1 binding has not been experimentally measured; however, binding of c-Jun and its partner c-Fos has shown evidence of cooperativity^40^. Similar studies examining dynamic p53 gene expression have modeled a modest cooperativity of *n* = 1.8^39^. As such, we chose to examine two potential Hill coefficients (*n* = 1.8 and *n* = 4). To estimate optimal parameters of mRNA stability and the value of *K*, we performed a parameter scan across 200 values of *α*_m_ from 0.02 to 1.4 h^−1^, approximating mRNA half-lives from 30 min to 36 h. Simultaneously, we also scanned 200 values of *K* from 0.01 (*K* « pJun) to 3 (*K* > pJun) *C*_s_ for each cluster and computed the RMSE. The local minima across surface plots were used to identify the estimated optimal parameters for both *n* = 1.8 (Fig. 7A) and *n* = 4 (Fig. 7B). Overall, trends in estimated decay rates remained similar to the mass-action model with clusters S2, T5, P3, and P5 estimated as long-lived, while the other clusters were comparatively estimated as less stable with higher decay rates. However, we did observe that many clusters exhibited variable *K* values, suggesting differences in relative affinity. This was most notable for cluster S2, which exhibited a higher *K* for both Hill coefficients, suggesting a low-affinity promoter.

In order to determine whether modeling gene expression through Hill equations improved the overall fit of the model, we compared the mass-action model and both Hill models to the experimental RNA-seq expression (Fig. 7C–E) and computed the RMSE for each cluster (Fig. 7F). For the most part, the Hill models provided minimal benefit compared to the mass-action model. However, clusters T1 and T3 both exhibited lower RMSE values, suggesting that, for some clusters, inclusion of promoter affinity can improve the model fit. As a final method, we tested whether incorporation of a delay term into the target gene expression (Eq. 4 or Eq. 8, respectively) could further improve the model (Supplementary Fig. 6A). We specifically tested all three models (mass-action, Hill *n* = 1.8, and Hill *n* = 4) across 25 delay values from 0.01 to 1 h and calculated optimal parameters based on minimization of RMSE (Supplementary Fig. 6B). Overall estimated decay rates between delay and non-delay models were largely similar. Similarly, for most of the gene clusters, incorporation of a delay had relatively modest impacts on RMSE (Supplementary Fig. 6C), and most clusters aligned best with relatively short delays. However, clusters T1 and T3 both showed benefits of delayed incorporation of nearly 1 h, suggesting potential specific regulation of these two clusters.

## Discussion

The JNK signaling pathway plays a pivotal role in cellular responses by balancing the induction of cell survival and cell death^16,41^. The temporal dynamics of JNK activation have previously been shown to promote distinct cellular fates across multiple stimuli, including UV light, oxidative stress, and endoplasmic reticulum stress^16,17,19–21^. Within our studies, we have found that these dynamics vary not only across various stimuli but also among individual cells exposed to the same stimulus, as cells exhibit variability in the duration or pulses of JNK activation over time. This variability within cells likely stems from variable levels of positive and negative regulators, similar to the findings from the Aoki lab, where variations in p38 and DUSP1 levels contributed to heterogeneity in JNK activation^19^. However, how these dynamics from previous studies ultimately contribute to downstream gene expression patterns has been unclear.

Our study demonstrates that variations in the duration or pulse numbers of JNK activity can elicit unique gene expression profiles. Similar to previous work examining p53 and NF-κB, these dynamics appear to be influenced in part by mRNA stability^6,12,42^. However, mRNA stability alone appears insufficient to explain all the observed gene expression patterns. Modeling gene expression as a Hill function improves the fit of some clusters, suggesting an additional layer of regulation may be promoter affinity. However, other mechanisms of regulation are also a possibility. For example, some of the observed gene expression patterns may rely on transcriptional cross-talk by additional transcription factors that may or may not be regulated by JNK. Consistent with this possibility, many of the observed genes were associated with NF-κB activity. Previous studies looking at the decoding of signals through PAMPs have suggested an array of combinatorial networks that drive gene expression patterns^3^. Included among these identified networks is a coherent feedforward loop regulated by both NF-κB and AP-1, as well as coordinated cross-talk between NF-κB and p38^3^. It is well established that anisomycin activates both JNK and p38, potentially providing another route for coordinated regulation of some of our observed gene expression patterns. In order to examine whether these other clusters rely on coordination between these pathways, a more in-depth characterization of *cis*-regulatory elements in the promoters of these genes, as well as characterization of NF-κB and p38 dynamics, is needed, which we aim to undertake in future studies. One additional mechanism that may explain clusters that do not behave as expected based solely on mRNA stability is the potential inhibitory phosphorylation of c-Jun. Recent work suggests that prolonged JNK activation hinders AP-1 activity by phosphorylation of c-Jun at threonine 91 and 93^43^. This mechanism may explain why sustained activation of JNK does not drive a continuous increase in gene expression, as we observed a gene cluster that exhibited transient upregulation with sustained JNK activation.

Similar to p53 and NF-κB expression patterns^6,7,12^, JNK dynamics appear to drive differential expression patterns of genes involved in specific pathways, such as cell death and inflammation-associated genes. These patterns may allow cells to differentially interpret stimuli. For example, genes that are rapidly and transiently induced may allow cells to reset and prepare for an additional stimulus, whereas genes that appear to accumulate over time, including cell death genes, may allow cells to integrate sustained signals until a critical threshold is met to induce cell death. While we did not observe any major distinctions in cell death by flow cytometry, we did observe slight variations in cell cycle arrest depending on the dynamics of JNK activation. Future studies examining how the frequency of JNK activation contributes to these patterns may provide further elucidation of the differential functions of these dynamics.

Mechanistically, these studies point to a highly conserved mechanism of regulating gene expression patterns through a combination of upstream signaling dynamics and mRNA stability. However, limitations must be kept in mind when applying modeling approaches. While our model focuses on the overall dynamics of gene expression, we do not factor in relative promoter strength. Genes within each identified cluster show variable levels of expression, which may reflect differences in promoter strength. As such, this model, as designed, is not equipped to predict overall changes in gene expression. Another factor that may limit the applications of the model is the inherent variability in decay rates of proteins and mRNA across cell types. Even within the published datasets analyzed, substantial variability exists in some genes, which may cause their dynamics to vary from the predicted model. Furthermore, unlike p53 and NF-κB, JNK activation dynamics may have additional effects beyond^35–38^ solely transcriptional regulation, including regulation of biological function through phosphorylation of additional substrates and through localization to specific subcellular compartments^23,44–46^. Further study is needed to understand how these additional functions coordinate within our basic model of transcriptional regulation. Clinically, the targeting of protein dynamics has proven difficult, particularly for transcription factors; however, kinase targeting has been far more successful. As a result, understanding the function of these dynamics and potential avenues to manipulate them may have significant impacts on the potential treatment of human disease.

## Methods

### Cell lines and culture

hTERT-RPE1 cells were originally purchased through ATCC (CRL-4000) and were grown in DMEM/F-12 media (Gibco: Hygromycin B Solution (50 mg/ml), Catalog Number 10687010) supplemented with 10% fetal bovine serum (FBS; Corning), 100 U/ml penicillin, and 100 µg/ml streptomycin. The cultures were maintained in a humidified incubator at 37 °C with 5% CO_2_. RPE1-hTERT cells expressing H2B-mCerulean and JNKKTR-mClover were generated via lentiviral transduction and selection with hygromycin and puromycin, respectively. Following selection, single-cell clones were generated by serial dilution and screened before imaging. All imaging studies were performed using clonally derived cells to minimize impacts due to genetic differences.

### Induction of JNK dynamics

To induce JNK activation, cells were treated with 50 ng/ml of anisomycin. For sustained activation of JNK, cells received a single treatment with anisomycin. To induce transient activation, cells were treated with anisomycin for 1 h followed by three washes with DMEM/F12 growth media and then placed into fresh media. Pulsed activation of JNK was induced by introducing a second anisomycin treatment at 4 h followed by an additional washout 1 h later as performed for transient conditions. Additional treatments were performed using sorbitol (Fisher, BP439–500), tunicamycin (Fisher, 35-161-0), thapsigargin (Fisher, 11-381), H_2_O_2_ (Sigma-Aldrich, H1009-100ML), TGF-β1 (Fisher, GF346), and TNFα (Fisher, 501615266) at the indicated concentrations.

### Live-cell microscopy

Live-cell imaging was performed using a Nikon Ti2 widefield fluorescence microscope equipped with a Tokai-Hit environmental chamber for gas and humidity control. Images were captured every 10 min using a Plan Apo λD ×20/0.8 NA objective. Excitation of fluorescent proteins (H2B-Cerulean and JNKKTR-mClover) was performed using a Spectra III light engine (CFP, 440 nm; YFP, 514 nm) with LED intensity set at 25%. CFP light was passed through a CFP/YFP/mCherry - Pinkel Triple excitation filter and 474/29 excitation filter. YFP excitation light passed through the same Pinkel Triple filter followed by 544/24 emission filter. Images were captured using an OrcaFusion BT camera on 16-bit image settings to capture H2B-mCerulean (200 ms exposure) and JNKKTR-mClover (300 ms exposure). Images were exported as individual tiff images for each time point and position for subsequent analysis.

### Quantification of kinase C/N ratio

Quantification of live-cell microscopy was performed using MATLAB p53Cinema^47^. Briefly, cells were segmented and tracked using captured H2B-mCerulean images with a minimum of 50 individual cells tracked per condition. Identified centroids were used to measure the mean fluorescence intensity of JNKKTR mClover within the nucleus of identified cells. Cytoplasmic-to-nuclear ratios (C/N ratio) were quantified by expanding the identified nucleus by three pixels to generate a cytoplasmic ring to approximate cytoplasmic fluorescence, as has been used in other studies^18^. The ratio between the cytoplasmic ring and nuclear measurements gave the C/N ratio. For quantification of pulse numbers, we used the findpeaks function in MATLAB with the assignment of a minimal peak threshold of 50% above average JNK activity at baseline to avoid spurious peak detection due to noise in individual cells. Quantification of overall durations of JNK activity was performed by calculating the duration of JNK activity above this same threshold.

### Western blot analysis

Cells were grown on 6 cm plates and treated according to the induction (sustained, transient, or pulsed) method. After treatment, cells were washed with 1× phosphate-buffered saline (PBS) after aspirating the culture medium. Cells were collected by scraping in 1 ml PBS and transferred to microcentrifuge tubes. An additional 1 ml of PBS was used to collect remaining cells. The collected cells were centrifuged at 13,000 rpm for 5 min, lysed in lysis buffer (ThermoFisher, 89900) containing protease and phosphatase inhibitors (ThermoFisher, 78440), and incubated on ice for 30 min with periodic agitation. Insoluble debris was removed by centrifugation at 13,000 rpm for 10 min at 4 °C. Protein concentration was determined by Bradford assay (Biorad, 5000006) using a standard curve of known BSA concentration.

Proteins (20 µg per lane) were separated on 4–20% Tris-Glycine gels (Biorad, 4561096) and transferred to PVDF membranes (Millipore, IPFL00010). Membranes were blocked in Licor Blocking Buffer (Licor, 927-70001) for 1 h and incubated overnight at 4 °C with primary antibodies specific to the target proteins. Primary antibodies include c-Jun (CST#9165; 1:1000), Phospho-c-Jun serine 73 (CST#3270, 1:1000), and β-actin (CST#4970,1:3000). After washing three times with 0.1% PBST, membranes were incubated with IRDye secondary antibodies (Licor) at a 1:5000 dilution for 1 h, washed again, and imaged using a Licor Odyssey Fc imaging system. All experiments were performed using three biological replicates.

### RNA sequencing

Cells were grown on 6 cm plates and treated according to the induction method (sustained, transient, or pulsed JNK activation). Additionally, for RNA sequencing we included an additional group that received treatment with the JNK inhibitor tanzisertib (10 µM) in addition to sustained anisomycin treatment. Total mRNA was isolated using the Qiagen RNeasy Mini Kit (Qiagen, 74106) at the indicated time points (0, 2-, 4-, 6-, or 8-h post-treatment). These time points were chosen to lag peak transcription factor activity by approximately 1 h based upon similar dynamic studies examining dynamic p53 transcriptional responses where peak mRNA lagged by about 1 h^7,12^. RNA was provided to the SDSU Genomics Sequencing Core Facility for quality assessment, library preparation, and sequencing. Libraries were prepared using the Illumina TrueSeq mRNA, and 45 samples were prepared for NExtSeq500 (1×75 bp) high-output reads. Sequencing was performed using three runs to get sufficient reads for all samples and was performed by the SDSU Genomics Sequencing Core Facility. All bioinformatic analyses were done using CLC Genomics Workbench (vs 24.0). Trimmed reads were mapped to the human genome vs hg38 as reference using a minimum length fraction of 0.8 and a minimum similarity fraction of 0.8. Differential expression was calculated on a gene basis using an absolute fold change cut-off of 2 and an FDR-adjusted *p* value of 0.05.

### Ordinary differential equation (ODE) modeling of JNK signaling

In order to model predicted gene expression dynamics, we designed an ODE model using MATLAB based on seven species. These seven species and the equations used are presented in Table 1. To model JNK activation, we used a constant (*c*), which we varied between 0 and 1 as a step stimulus. Estimates of rate parameters were pulled from literature or based upon p53 dynamic models^10,11,26–33^. For modeling variations in mRNA stability, we varied the mRNA decay rate (*α*_m_). *C*_s_ denotes simulated concentration units. Global sensitivity analysis was performed using the SimBio toolbox in MATLAB, and Sobol indices were computed using the Saltelli method^48^ across the listed parameter values in Table 2 and 1000 samples. For alignments of models and experimental measures, RMSE was computed using the RMSE function in MATLAB using a linear interpolation to fill in sparse datapoints from RNA-seq timepoints. We deliberately avoid non-linear interpolations like spline fitting due to the sparse nature of the timepoints in the RNA-seq dataset. For computations of decay rates, it was assumed that *α*_m_ values that minimized RMSE were the optimal decay rate. Similar strategies were used to estimate both decay rates and *K* values for modeling Hill-equation regulation of target gene expression. Delay differential modeling was performed using the same equations with the introduction of a lag term into Eq. 4. A delay parameter scan was performed from 0.01 h to 1 h across 25 uniform steps. The model was run using the dde23 solver in MATLAB.

### K-means clustering

K-means clustering of DEGs was performed using MATLAB. Briefly, differential expression analysis from CLC was imported into MATLAB, and significantly DEGs were identified based on an FDR-adjusted *p* value < 0.05, an absolute Log_2_Fold Change >1, and a Max Group Mean >1 and restricted to protein-coding transcripts. Data was organized based on treatment condition and timepoint, then rescaled between 0 and 1. K-means clustering was then performed on each condition (sustained, pulsed, or transient) using the k-means function with the default seed. Selection of the cluster numbers was determined using combinations of the silhouette score, the elbow method, and minimizing variations in cluster size.

### Cell cycle analysis

In all 3.5 × 10^5^ hTERT-RPE1 cells were plated onto 6-cm plates (Genesee) and allowed to grow for 24 h. At the time of treatment, the media was replaced with fresh media, and treated cells received anisomycin treatments as previously outlined. After 24 or 48 h, the media were collected and placed into labeled tubes. Cells were trypsinized and added to the collection tube containing the original media. Cells were spun down at 400 × *g* for 4 min and washed twice with PBS to remove residual media. Cell pellets were re-suspended in 300 μl PBS and passed through a 45 μm cell strainer to remove clumps. Cells were then fixed by the addition of 700 μl cold ethanol dropwise. Fixed cells were stored at −20 °C until staining. Prior to staining, cells were spun down at 750 × *g* for 5 min and washed twice with PBS to remove ethanol. In all, 1 × 10^6^ cells were then re-suspended in 250 μl PBS + 10 μl propidium iodide and 5 μl RNaseA. Cells were incubated at 37 °C for 30 min, then brought to 500 μl final volume by PBS and passed through a 45 μm cell strainer prior to analysis on a BD Accuri C6. Single cells were identified based on initial gating on forward and side scatter measurements, followed by PI-A and PI-H gating. Gates for sub-G1, G1, S, and G2/M phases were manually annotated to histograms of the PI stain. Data for cell cycle distributions were presented as the mean ± SEM of three biological replicates.

## Supplementary information

CompiledSupplementalFiguresR2

SupplementalData 1

## Data Availability

Single-cell KTR traces used within this study have been uploaded to Mendeley Data with unique identifiers. The basic JNK signaling model has also been uploaded to Mendeley Data. RNA-seq data were uploaded to GEO; JNKKTR in response to multiple stimuli (Hanson, Ryan (2025), “JNK dynamics in response to multiple stimuli”, Mendeley Data, V2, 10.17632/4s278fpzp4.2); Manipulation of JNK dynamics by Anisomycin (Hanson, Ryan (2025), “Manipulation of JNK dynamics by Anisomycin”, Mendeley Data, V1, 10.17632/sjkzrs3sfz.1); JNK model of dynamic gene regulation (Hanson, Ryan (2025), “JNK model of dynamic gene regulation”, Mendeley Data, V1, 10.17632/h828r74cjt.1); RNA-seq data: GEO (GSE298022).
