# Supplementary material for: JNK activation dynamics drive distinct gene expression patterns over time mediated by mRNA stability
Source: NPJ Syst Biol Appl. 2025 Oct 21;11:117. doi: 10.1038/s41540-025-00590-2 (PMC12540708; doi:10.1038/s41540-025-00590-2)
Supplement: Supplementary file 1 — CompiledSupplementalFiguresR2 [file 41540_2025_590_MOESM1_ESM.pdf]

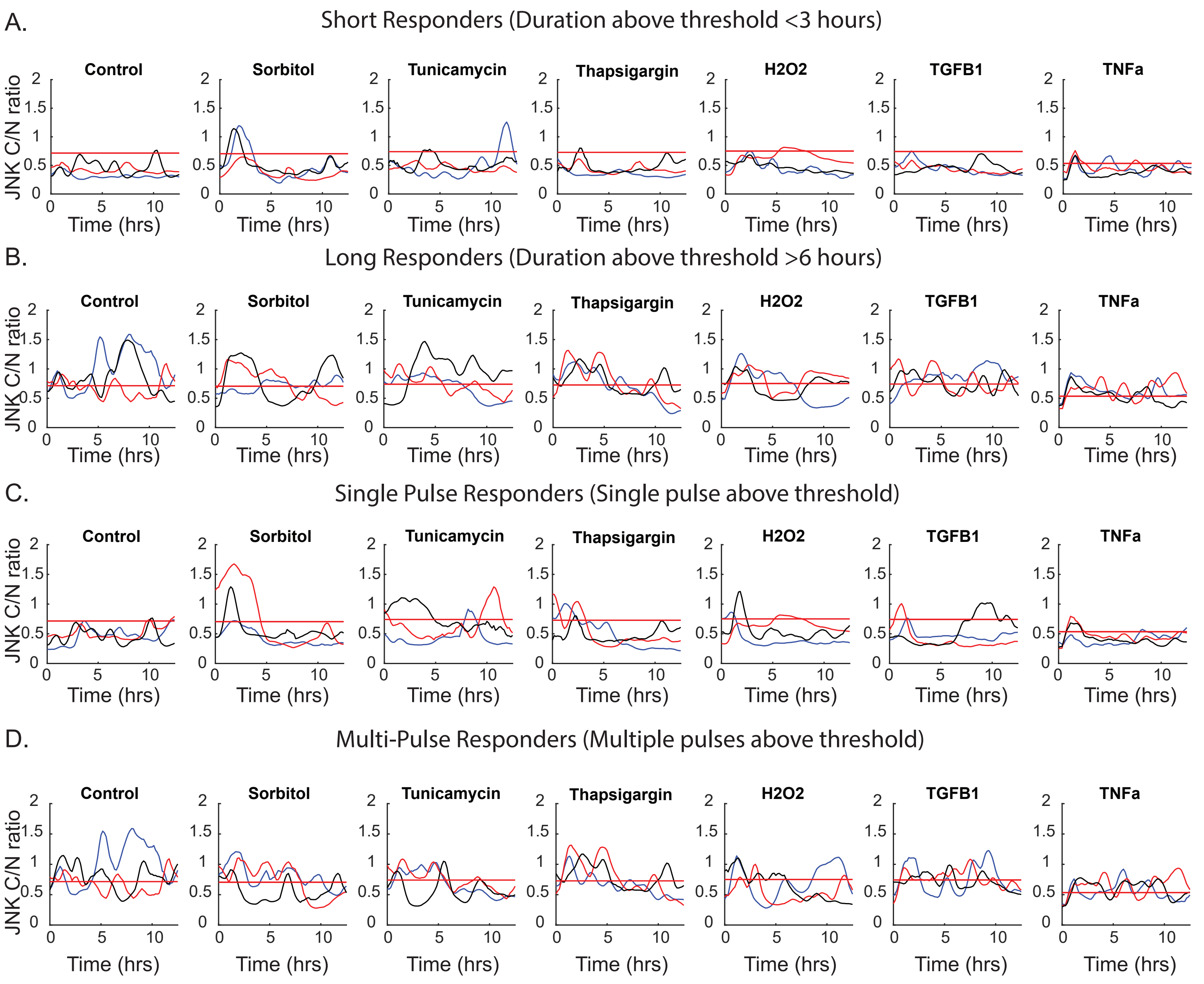

Supplementary Figure 1: JNK dynamics vary dramatically from cell to cell
A-D) Individual traces of single cells showing JNK activity (C/N ratio) of either short duration (**A**), long duration (**B**), singular pulses (**C**), or multiple pulses (**D**).

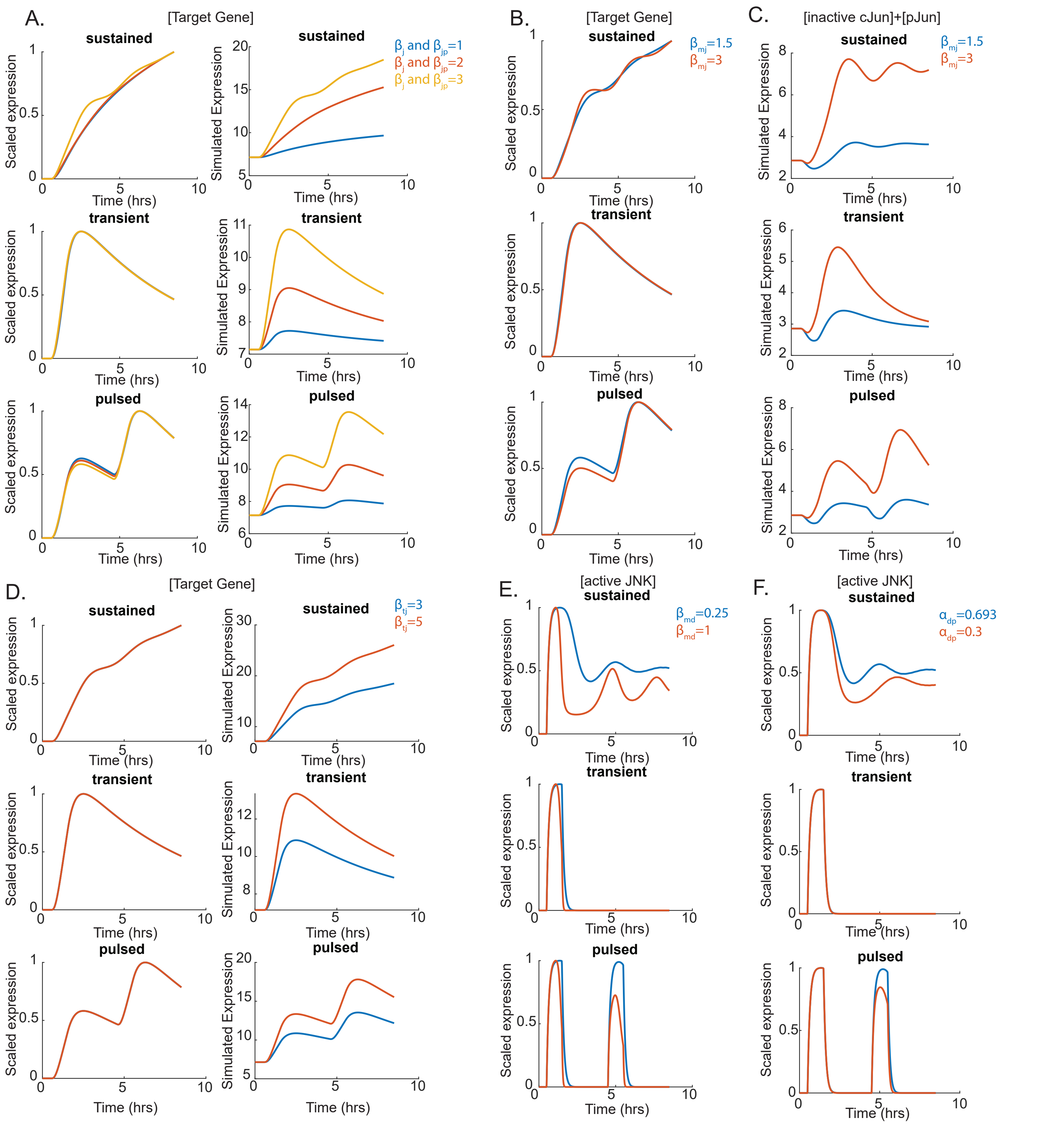

Supplementary Figure 2: Impacts of varying production and decay rates on model outputs
A)Varying production rates of inactive cJun (β_j) or pJun (β_{jp}) had minimal effects on the scaled expression dynamics of target genes but influence simulated concentration. **B)** Varying cJun autoregulation had minimal impacts on scaled expression of target genes but alters simulated levels of total cJun (**C**). **D)** Production rates of target genes (β_{tj}) does not impact the shape of gene expression but does influence absolute simulation concentration. **E-F)** Altering either production of DUSP1 mRNA (**E**) or stability of DUSP1 protein (**F**) dampens modeled JNK activity in sustained and pulsed conditions.

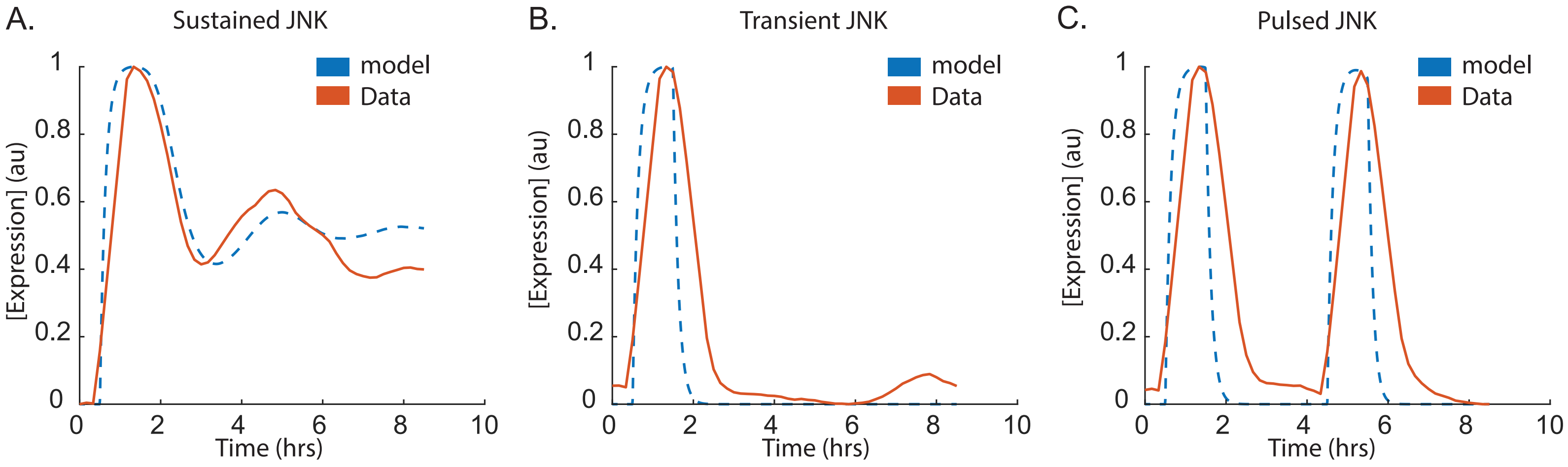

Supplementary Figure 3: ODE model aligns similarly to JNKKTR traces
A-C) Scaled ODE traces (dashed line) for active JNK were compared to scaled JNK C/N ratios from Figure 2 (solid orange line) for sustained (**A**), transient (**B**), or pulsed (**C**) stimulation. Data plotted as relative expression from 0-1.

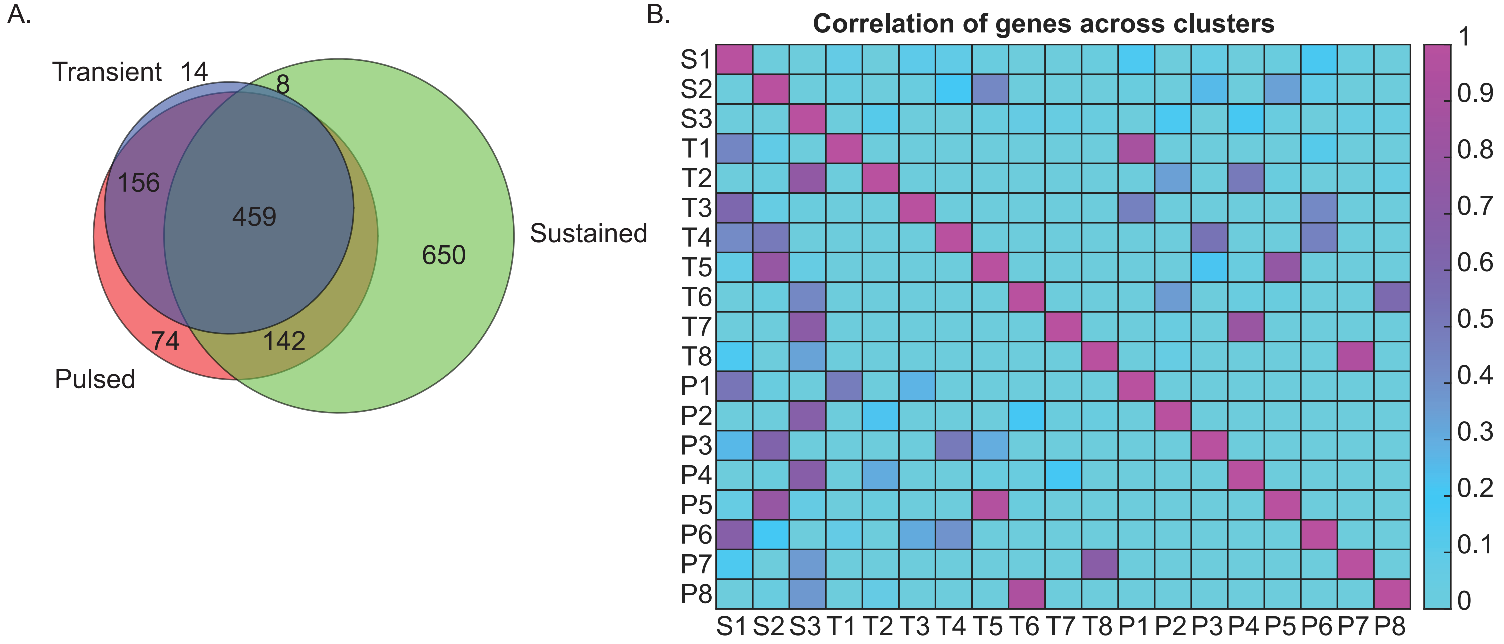

Supplementary Figure 4: Gene dynamics regulated by JNK overlap with other clusters
A)Venn diagram of differentially expressed genes across sustained, transient, or pulsed conditions highlights strong overlap in gene networks. **B**) Cross-correlation matrix identifying overlaps in genes identified across clusters demonstrates specific clusters tend to correlate strongly with others when JNK dynamics vary.

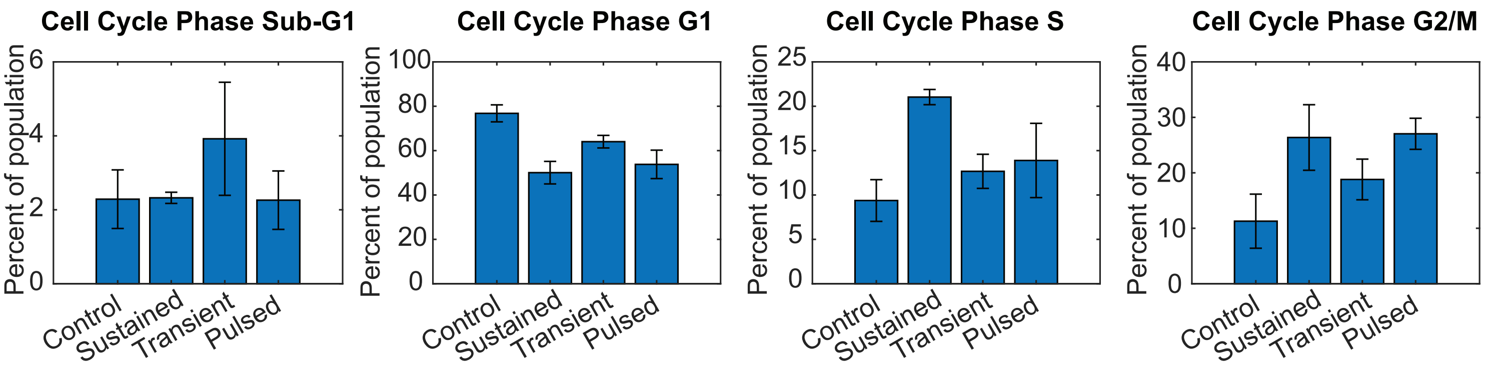

Supplementary Figure 5: Anisomycin-induced JNK dynamics have modest impacts on cell cycle progression.
Cell cycle analysis of either control cells or cells undergoing sustained, transient, or pulsed activation. Average percentage of the population±SEM for subG1, G1, S, and G2/M phases are plotted.

A.

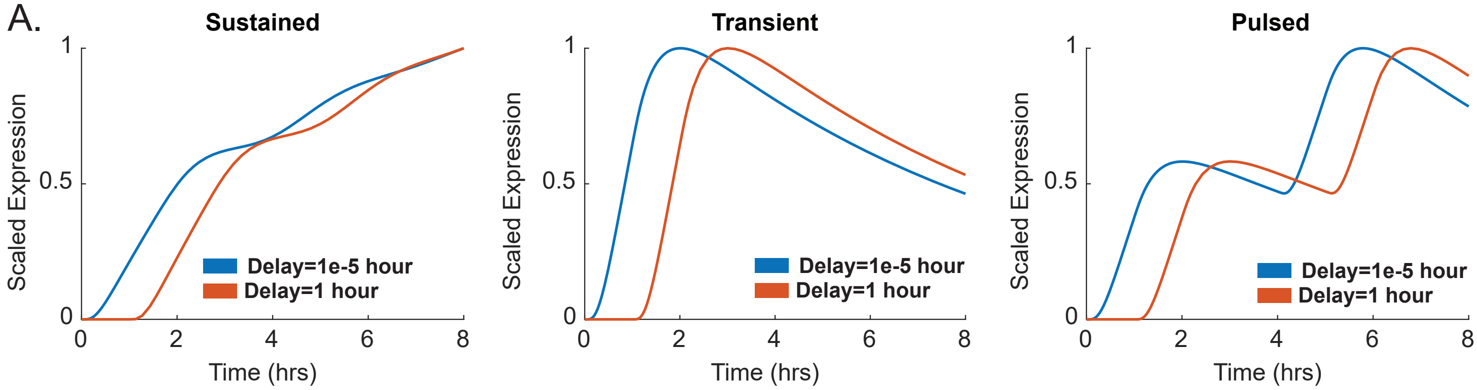

B.

Mass-Action w/Delay

Cluster	Estimated mRNA decay rate (α_m) h^{-1}	Delay (hr)
S1	0.21	0.01
S2	1.4	0.13
T1	0.14	1.00
T3	0.02	1.00
T4	0.20	0.26
T5	0.60	0.13
P1	0.02	0.55
P3	0.36	0.13
P5	1.13	0.51
P6	0.11	0.05

Hill n=1.8 w/Delay

Cluster	Estimated mRNA decay rate (α_m) h^{-1}	Estimated K (C_s)	Delay (hr)
S1	0.37	0.01	0.01
S2	1.05	3.00	0.01
T1	0.23	0.01	0.84
T3	0.02	3.00	0.30
T4	0.23	0.09	0.01
T5	0.96	0.10	0.05
P1	0.21	0.01	0.13
P3	0.51	0.09	0.01
P5	1.39	0.12	0.18
P6	0.45	0.01	0.01

Hill n=4 w/Delay

Cluster	Estimated mRNA decay rate (α_m) h^{-1}	Estimated K (C_s)	Delay (hr)
S1	0.38	0.01	0.01
S2	0.41	2.53	0.01
T1	0.19	0.01	0.59
T3	0.03	2.74	0.30
T4	0.19	0.12	0.01
T5	0.76	0.12	0.01
P1	0.19	0.01	0.05
P3	0.48	0.09	0.01
P5	1.23	0.09	0.05
P6	0.47	0.01	0.01

C.

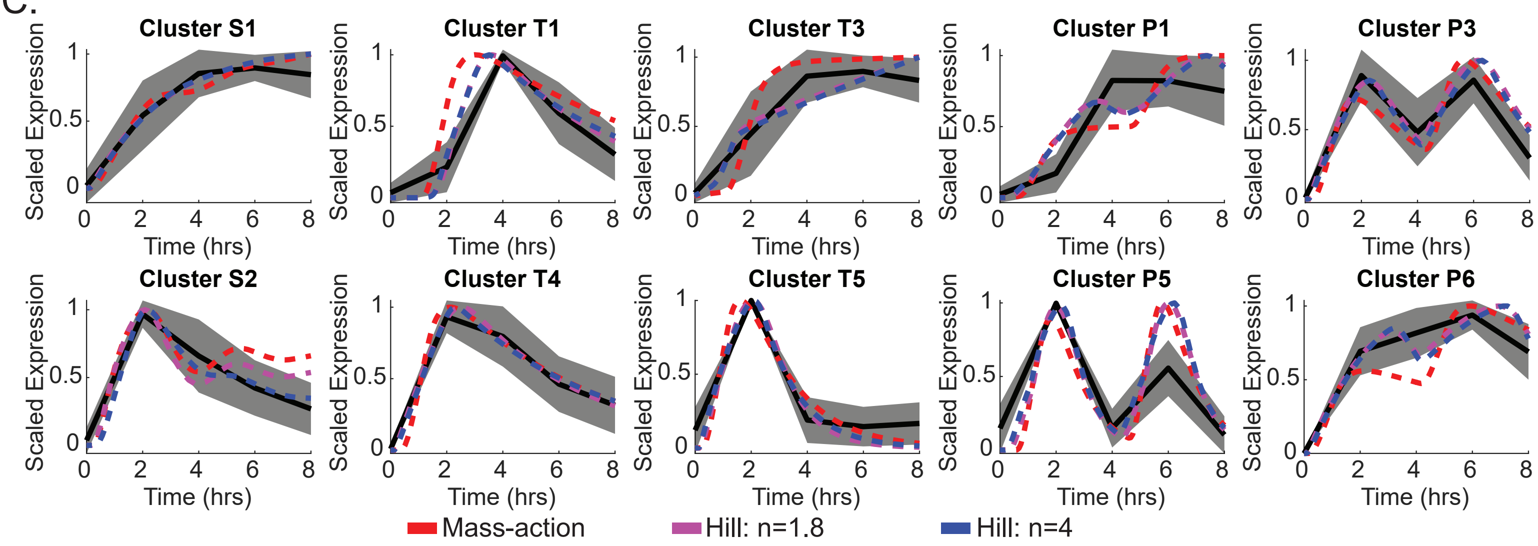

Model	Cluster S1 RMSE	Cluster S2 RMSE	Cluster T1 RMSE	Cluster T3 RMSE	Cluster T4 RMSE	Cluster T5 RMSE	Cluster P1 RMSE	Cluster P3 RMSE	Cluster P5 RMSE	Cluster P6 RMSE
Mass-action	0.059	0.146	0.200	0.137	0.087	0.132	0.198	0.121	0.212	0.181
Hill coeff 1.8	0.049	0.118	0.101	0.069	0.038	0.109	0.145	0.088	0.192	0.086
Hill coeff 4	0.051	0.088	0.104	0.072	0.041	0.099	0.144	0.082	0.184	0.104

Supplementary Figure 6. Delay models modestly improve fit
A) Example plots of scaled gene expression from sustained, transient, and pulsed models with very short delay (blue) or 1 hour delay(orange). **B**) Estimated optimal mRNA decay rates, delays, or K-values based on parameter scans across all three models. **C**) Plots of RNA-seq gene clusters in comparison to Mass-Action (red), Hill function n=1.8 (magenta), and Hill function n=4 (blue) using parameters defined in (**B**). Lower panel shows the RMSE between each model and experimental data.

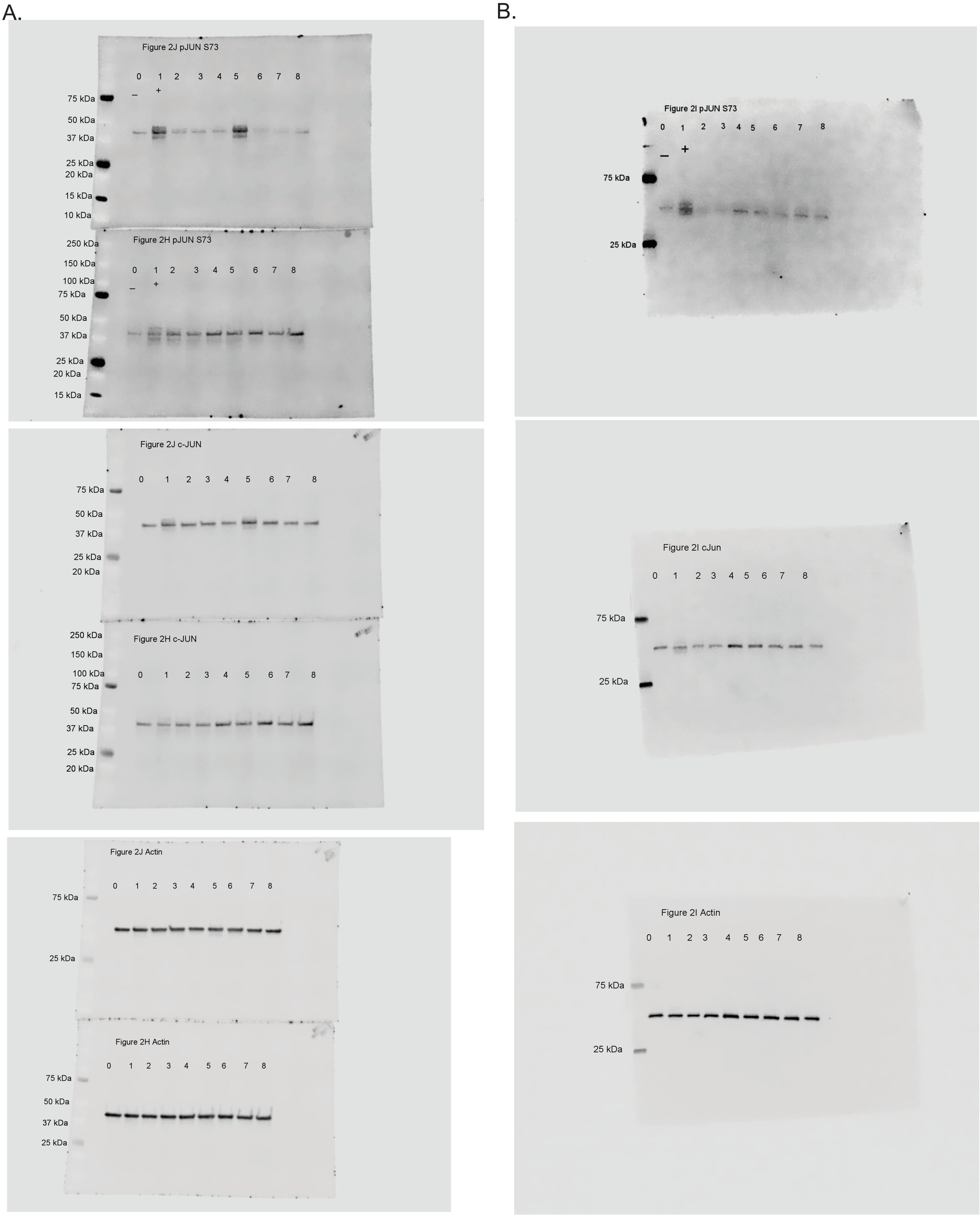

Supplementary Figure 7: Original Licor images of western blots presented in Figure 2H-J.
A)Original western blots for Figures 2H (top blots) and 2J (bottom blots) for pJUN S73 (top), c-Jun (middle), and Actin (bottom). Ladder sizes indicated where visible. - and + indicate first addition of anisomycin which induces c-Jun phosphorylation. Lanes indicated as they appear in Figure 2. **B**) Original western blot images for Figure 2I for pJUN S73 (top), c-Jun (middle), and Actin (bottom). Ladder sizes indicated where visible.
- and + indicate first addition of anisomycin which induces c-Jun phosphorylation. Lanes indicated as they appear in Figure 2.

Supplementary Data 1: Genes within each identified cluster

Supplementary Data table provides the list of genes within each gene cluster identified in Figure 4
